# Exosome-like nanovesicles from *Dunaliella salina* efficient sequential Co-delivery of anti-PDL1 and miR-375 for enhancing gene/immune therapy

**DOI:** 10.1016/j.ncrna.2025.08.007

**Published:** 2025-09-01

**Authors:** Zhaoyi Wei, Mengxi Zhu, Shan Li, Junling An, Yiwen Liu, Shuying Feng, Tingting Yang, Shegan Gao, Gaofeng Liang

**Affiliations:** a Henan International Joint Laboratory of Small Nucleic Acid and Tumor Precision Theranostics, School of Basic Medicine and Forensic Medicine, Henan University of Science & Technology, Luoyang City, Henan Province, 471023, China; b Institute of Organoids on Chips and Drug Translational Research, Henan Academy of Sciences, Zhengzhou City, Henan Province, 450009, China; c Clinical Medical College Henan Key Laboratory of Microbiome and Esophageal Cancer Prevention and Treatment, The First Affiliated Hospital of Henan University of Science and Technology, Luoyang City, Henan Province, 471023, China; d Clinical Medical College, Henan University of Chinese Medicine, No. 156, Jinshui East Road, Zhengzhou City, Henan Province, 450046, China

**Keywords:** Exosome-like nanovesicles, *Dunaliella salina*, anti-PD-L1, miR-375, Esophageal cancer, Drug delivery

## Abstract

**Background:**

Esophageal cancer is one of the common malignant tumors of digestive system. Despite many advances in the treatment of esophageal cancer, many challenges remain. As an endogenous extracellular vesicle, exosomes are increasingly presenting their immense potential in drug delivery. However, it remains a bottleneck to obtain a large quantity of uniform, stable, and multi-component controllable exosomes with low cost and time.

**Methods:**

A novel targeted drug delivery system based on exosome-like nanovesicles has been developed using the natural Marine single-celled salt *Dunaliella salina* (DENV) to conjugate c (RGDyK) peptide on its surface to achieve targeted drug delivery to esophageal cancer cells. In addition, miR-375 was loaded into cRGD-DENV by electroporation and aPD-L1 was coupled to its surface by matrix metalloproteinase-2 (MMP-2). Characterizations were performed to confirm the successful preparation of engineered exosomes. The effects of engineered exosomes on tumor cell viability, migration, invasion and apoptosis were examined *in vitro* and the effects of engineered exosomes on esophageal cancer cells were further verified *in vivo*.

**Results:**

The engineered DENV delivery system was prepared and characterized. It exhibited a uniform particle diameter (approximately 150 nm) with *in vitro* sustained release features in the presence of MMP-2/9. Importantly, the cRGD-DENV was effective, promoted selective delivery of cargoes to the tumor site, and reduced nonspecific uptake of the DENV cargoes, significantly inhibiting tumor growth *in vitro*. *In vivo* results showed that cRGD-DENV-aPDL1/miR375 significantly inhibited tumor growth and affected the proliferation, migration and invasion of esophageal cancer cells by regulating YWHAZ.

**Conclusions:**

The potential of *Dunaliella salina* exosome-like nanovesicle carrier delivery system in cancer therapy and can provide a very promising platform for the rapid and large-scale generation of functionalized exosome-like nanovesicles.

## 1 Introduction

Exosomes, known as “Trojan-Horse” [1], are naturally transported small membrane vesicles (40–150 nm) originating from endocytosis, have been used to facilitate intracellular delivery of multiple varied cargoes, and are closely associated with many physiological or pathological processes [2]. In recent years, the numerous peer-reviewed publications and the explosion of new exosome-based biotechnology businesses focusing on manipulating and utilizing exosomes as biomarkers, vaccines, drug carriers, or novel therapeutics have demonstrated the considerable interest of many researchers in this field [3,4]. Exosomes increasingly present great potential in drug delivery due to nanoscale dimensions, minimal immunogenicity, and low toxicity [5]. For example, exosomes derived from mammalian cells can serve as efficient drug-delivery vehicles. These exosomes can encapsulate various drugs, such as doxorubicin (DOX), paclitaxel, and curcumin, among others, for the treatment of tumors [6]. However, exosomes sourced from mammalian cells are limited in clinical application due to the substantial cost of mass production, the time-consuming nature of the production process, and the issue of uncontrolled exosome contents [7]. Therefore, it is urgent to develop safe alternative sources of exosomes to solve the problem that limits their further application.

Recently, exosome-like nanovesicles derived from plants have garnered considerable attention due to the merits of high yields, low production costs, and low cytotoxicity [8]. Inspired by this, *Dunaliella salina* (*D. salina*), a single-celled halophilic eukaryotic algae without cell walls, was selected to prepare exosome-like nanovesicles [9]. The *Dunaliella salina* is mostly oval or elliptical in shape. It has no fibrous cell wall and its appearance can easily change with the environment. The protein nucleus and starch granules also vary depending on the environment [10]. As a photosynthetically autotrophic organism, *D. salina* grows fast, is easy to cultivate, and is rich in essential glycerol and β-carotene with nutrition [11]. More importantly, *D. salina* can be grown on a large scale in seawater, which means that we can obtain exosome-like nanovesicles at a lower cost and shorter production time compared with mammalian cells [12,13]. Natural exosomes, derived from endogenous cells, possess inherent biocompatibility and low immunogenicity but are limited by low yield, heterogeneous cargo, and weak targeting ability [14]. In contrast, engineered *Dunaliella salina*-derived vesicles (DENV) offer advantages such as scalable production, controllable cargo loading (e.g., miR-375), and enhanced targeting via surface modification (e.g., cRGD conjugation) [15]. While natural exosomes benefit from natural cell-recognition properties, engineered DENV exhibit superior functional tunability, making them more suitable for tailored therapeutic applications. Therefore, *D. salina* can be used as a promising bioreactor, offering a potential solution to the challenge of large-scale exosome preparation for targeted drug delivery.

Esophageal cancer is a serious disease that poses a significant threat to human health worldwide, with its incidence and mortality rates remaining high [16]. KYSE-150 cells are well-established as a representative model for esophageal squamous cell carcinoma (ESCC). This cell line, derived from a primary ESCC tumor, exhibits key pathological features of aggressive disease, including high proliferative capacity (13.7-h doubling time) and characteristic genetic aberrations (e.g., p16 silencing via CpG methylation), which align with clinical ESCC profiles [17]. Furthermore, the esophageal squamous cell carcinoma cell line KYSE-150 has a moderate growth rate and moderate invasiveness. At the genetic level, in addition to the common alterations found in ESCC, it also has a unique PI3K/AKT pathway mutation, which distinguishes it from cell lines with changes in NOTCH1 or FGFR1 [18]. Notably, KYSE-150 has been a widely used cell line in ESCC research, including studies on therapeutic response and nanoparticle-mediated interventions, validating its relevance for investigating tumor biology and treatment efficacy [19].

MiRNA is an 18–23 nt noncoding RNA, affecting the occurrence, development, metastasis and drug resistance of cancer by targeting and regulating tumor-related genes [20]. miR-375 is frequently downregulated in ESCC and acts as a tumor suppressor by inhibiting YAP1, PI3K/AKT to suppress proliferation and invasion [21]. Tumor cell surface PD-L1 binding to immune cell PD-1 inhibits immune activity, enabling immune evasion; aPD-L1 blocks this interaction, relieving suppression and activating immune cells to recognize and kill tumors [22]. miR-155 suppressed tumor growth by inhibiting the expression of PD-L1. Therefore, by regulating the expression level of PD-L1 through miRNA and combining with aPD-L1 treatment, a synergistic effect can be achieved to enhance the anti-tumor effect [22]. Furthermore, some stimulatory responsive biomaterials have significant implications for immunotherapy in regulating the tumor microenvironment, providing important insights for our research [[23], [24], [25]].

**Fig. 1:**
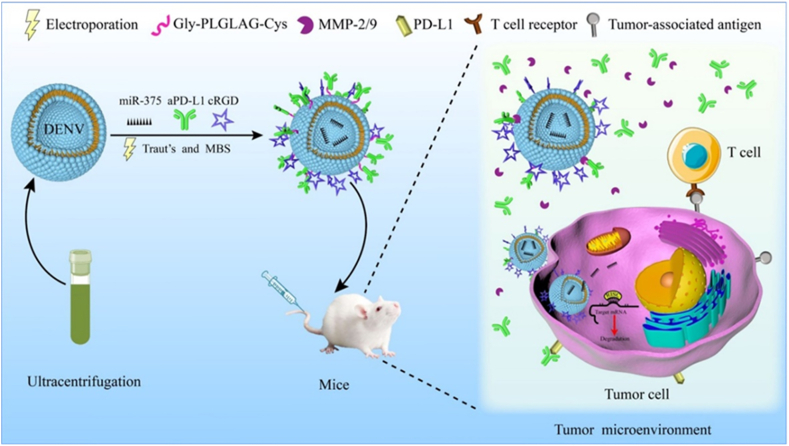
Schematic design of the targeted drug delivery system based on DENV and its regulation strategy.

In this study, we innovatively used natural single-cell *D. salina* to prepare exosome-like nanovesicles (DENV). To improve its targeting capability, the c(RGDyK) (cyclo (Arg-Gly-Asp-D-Tyr-Lys) peptide, a specific tumor-homing polypeptide, was conjugated to the DENV surface to construct engineered exosomes (cRGD-DENV) to facilitate targeting cellular uptake through α_v_β_3_ integrin receptor-mediated endocytosis in tumor cells and tissue [26]. Ultracentrifugation was used to obtain a substantial quantity of exosome-like nanovesicles and a transmission electron microscope (TEM) was utilized to characterize them. Then we proved that cRGD-DENV can be specifically taken up by tumor cells, and near-infrared fluorescence (NIRF) imaging indicated that the cRGD-DENV efficiently targets tumor sites after intravenous injection, suggesting cRGD-modified DENV could be a promising nano-platform for tumor-targeted therapy. Furthermore, miR-375 was loaded into the cRGD-DENV by electroporation, and the Gly-PLGLAG-Cys peptide (an MMP-2 sensitive peptide), which can be cleaved by the highly expressed MMP-2 in the tumor microenvironment (TME). MMP-2 sensitive peptide was used as a linker between aPD-L1 and DENV. The schematic of the targeted drug delivery system based on DENV is shown in Fig. 1. In this design, the delivery system can achieve the purpose of releasing aPD-L1 and miR-375 sequentially: the high expression of matrix metalloproteinase in TME can cleave the Gly-PLGLAG-Cys peptide, and the aPD-L1 was released from cRGD-DENV-aPD-L1/miR-375 when this delivery system entered into tumor surroundings. Subsequently, the released aPD-L1 binds to the PD-L1 on tumor cells to enhance anti-tumor immune response. Then miR-375 was delivered into tumor cells and post-transcriptional suppressed the YWHAZ gene, an oncogene in receptor cells. Moreover, we have further investigated the physiological role of engineered DENV derived from *D. salina* in esophageal cancer cells and the capability of drug delivery *in vivo*. The results demonstrated that exosome-like nanovesicles derived from *D. salina* are promising biomaterials for delivery with extensive, broad clinical applications.

## 2 Materials and methods

### 2.1 Materials

Fetal bovine serum was purchased from Wisent Biotechnology Co., Ltd. (Nanjing, China). Anti-PD-L1 (aPD-L1) was obtained from Invivogen (California, USA). The c(RGDyK) (cyclo(Arg-Gly-Asp-D-Tyr-Lys)) peptide and the Gly-PLGLAG-Cys polypeptide were synthesized by GL Biochem Co., Ltd. (Shanghai, China). 3-Maleimidobenzoic acid N-hydroxysuccinimide ester (MBS) and 2-Iminothiolane hydrochloride (Traut's Reagent) were purchased from Sigma-Aldrich (St. Louis, MO, USA). Annexin V-FITC Apoptosis Detection Kit was obtained from Sangon Biotech (Shanghai, China). Rabbit anti-caspase-9 antibody and anti-caspase-3 antibody were purchased from Boster Biological Engineering Co., Ltd. (Wuhan, China). Rabbit anti-CD3 antibody was obtained from Proteintech (Wuhan, China), and anti-CD4 antibody and anti-CD8 antibody were purchased from Abcam (Shanghai, China).

### 2.2 Cell culture and animals

The human esophageal cancer cell lines KYSE-150 were purchased from the Cell Bank of the Chinese Academy of Sciences (Shanghai, China). KYSE-150 cells were cultured in Roswell Park Memorial Institute-1640 medium (RPMI-1640) supplemented with 10 % FBS and 1 % penicillin-streptomycin at 37 °C in 5 % CO_2_ incubators.

The female Balb/c nude mice and Balb/c mice (18–20 g, 4–5 weeks old) were supplied by Nanjing Cavins Biotechnology Co., Ltd. and raised under 25 ± 1 °C with free access to food and water. All the animal experiments were carried out according to the guidelines approved by the ethics committee of Henan University of Science and Technology (Luoyang, China).

### 2.3 Isolation and purification of DENV

*D. salina* was cultured with PKS liquid medium (1.5 M NaCl, 10 mM KNO_3_, 50 mM NaHCO_3_, 5 mM MgSO_4_·7H_2_O, 0.4 mM KH_2_PO_4_, 2 μM FeCl_3_·6H_2_O, 5 μM EDTA, 7 μM MnCl_2_·4H_2_O, 1 μM CuCl_2_·2H_2_O, 1 μM ZnCl_2_, 1 μM CoCl_2_·6H_2_O, 1 μM (NH_4_)Mo_7_O_24_·4H_2_O, 185 μM H_3_BO_3_, 0.2 mM CaCl_2_) at the conditions (temperature 26 °C, light-dark ratio 14:10, light intensity 4000 Lux m^−2^ s^−1^). Subsequently, the culture medium was collected, and DENV was isolated using the ultracentrifugation method, which is detailed below. The detailed and specific steps are presented: (1) Centrifuge the culture supernatant at 2000×*g* at 4 °C for 20 min to remove the *D. salina* cells. (2) Retrieve the supernatant obtained from the preceding step and subject it to centrifugation at 10,000×*g* for 30 min at 4 °C to remove cell debris. (3) Take the supernatant from the previous step and centrifuge at 100,000×g at 4 °C for 90 min (4) Discard the supernatant and resuspend the DENV with PBS. Then, DENV was quantified by BCA assay and stored at −80 °C after aliquoting.

### 2.4 Preparation and characterization of DENV and cRGD-DENV-aPD-L1/miR-375

Firstly, chemical coupling technology was used to prepare cRGD-DENV-PLGLAG by coupling cRGD, Gly-PLGLAG-Cys peptide, and DENV. The fluorescence co-localization method was used to determine the coupling efficiency. In brief, 0.5 μg/mL DIO was added to labeled DENV, and cRGD peptide was labeled by Cy5.5-NHS, according to the manufacturer's instructions, to obtain Cy5.5-cRGD; this process was achieved by the reaction of the amino group with NHS, and the unreacted excess dye was removed through a purification column. Next, Cy5.5-cRGD (0.5 mg/mL) was mixed with an excess of Traut's reagent at room temperature for 30 min to generate sulfhydryl groups. Simultaneously, DIO-DENV was incubated with MBS (0.1 mg/mL) for 30 min to produce maleimide pendant groups on its outer membrane. After that, DENV was mixed with the thiolated cRGD and Gly-PLGLAG-Cys peptide solution for 1 h and obtained cRGD-DENV-PLGLAG. Then, the cRGD-DENV-PLGLAG was mixed with Traut's reagent. Then, the aPD-L1 was incubated with MBS at room temperature for 30 min. Finally, cRGD-DENV-aPD-L1 was obtained by mixing aPD-L1 with thiolated cRGD-DENV-PLGLAG above and removing free unreacted substances by ultracentrifugation. HPLC quantified the aPD-L1 loading efficiency (Agilent 1100, Germany).

The miR-375 plasmid was loaded using a typical electroporation method. Briefly, cRGD-DENV or cRGD-DENV-aPD-L1 (0.5 mg/mL) and miR-375 plasmid (0.1 mg/mL) were mixed into 1 mL conductivity buffer, respectively. The miR-375 plasmid was loaded by electroporation under 300 V and 250 μF conditions, and its loading efficiency was calculated by qRT-PCR, as described in our previous studies [27]. After loading, they were centrifuged at 100,000×*g* at 4 °C for 90 min to remove unbound miR-375 and then resuspended in PBS.

Take the suspension of DENV and cRGD-DENV-aPD-L1/miR-375 on a copper grid and leave them at room temperature for 5 min, respectively. Drop 3 % phosphotungstic acid solution on the copper mesh about 10 μL, and counterstain it for 5min. Bake with an incandescent lamp at 65 °C for about 15 min before taking pictures under the microscope and analyzing the morphology of each sample by a TEM. The size distribution of the DENV and cRGD-DENV-aPD-L1/miR-375 was determined by the Zetasizer Nano ZS instrument (Malvern, UK).

The sensitive properties of Gly-PLGLAG-Cys peptide to MMPs were confirmed by the release of aPD-L1 from cRGD-DENV-aPD-L1/miR-375 in PBS containing or not containing MMPs, as well as cell experiments. For determination, the equilibrium dialysis method was applied to detect aPD-L1. cRGD-DENV-aPD-L1/miR-375 solution (2 mL) was filled into the dialysis bag and immersed in 50 mL PBS (pH 7.4, containing MMPs or not). Then, the samples were shaken for 8 h at 37 °C at 120 rpm. At predetermined time points, the samples were collected for quantitative analysis by the HPLC system. Equipment model (Kejie, P1201), column type (C18, 5 μm, 250 × 4.6 mm), mobile phase composition (Acetonitrile: Water), flow rate (1 mL/min), detection wavelength (254 nm), and injection volume (20 μL). Also, we investigated whether pH (pH 7.4 and pH 6.5) and serum had an effect on the release of aPD-L1 from cRGD-DENV-aPD-L1/miR-375, and the method was the same as above. DIO and Cy5.5 fluorescent dyes were used for the cell experiment to label DENV and aPD-L1, respectively. After preparing cRGD-DENV-aPD-L1/miR-375, it was added to KYSE-150 cells to detect the release of aPD-L1 via a CLSM (ZEISS 880, Germany).

### 2.5 Cellular uptake

DENV (0.5 mg/mL) was briefly suspended in 100 μL of diluent and mixed with DiI (3 μL). After incubating at room temperature for 30 min, 1 % BSA (1 mL) was added to stop the labeling reaction, and the ultracentrifugation method was used to re-isolate the labeled DENV. When the confluence of KYSE-150 cells reaches 50 %, add DAPI (10 μL) to the glass-bottom Petri dish and incubate for 10 min, then wash with PBS. DiI-labeled DENV (5 μg) was added to KYSE-150 cells were observed during the period using a CLSM with a live cells workstation to examine the cellular uptake of DiI-labeled DENV at different time points.

### 2.6 Cell proliferation experiment

*In vitro*, cytotoxicity was measured by performing MTT assay on KYSE-150 cells. The cells (1 × 10^4^/mL) were inoculated into a 96-well plate (NEST, Wuxi, China) for 24 h; then incubated with different samples (Control, DENV, cRGD-DENV/miR-375, cRGD-DENV-aPD-L1, cRGD-DENV-aPD-L1/miR-375) for another 48 h. Thereafter, 10 μL MTT (5 mg/mL) was added to each well, and after incubating for another 4 h, DMSO (150 μL) was added to each well and shaken for 10 min to melt the crystal sufficiently. The absorbance of each well was determined at 490 nm on an enzyme-linked immunosorbent monitor, and the results were assayed.

### 2.7 Cell migration and invasion assay

The wound-healing assay was performed to study cell migration capacity as described previously. KYSE-150 cells were seeded into 6-well plates when they reached 70 %–80 % confluence; a scratch in the cell monolayer was made and then incubated with different samples (Control, DENV, cRGD-DENV/miR-375, cRGD-DENV-aPD-L1, cRGD-DENV-aPD-L1/miR-375). Finally, cell migrations were recorded at 0 h, 12 h, 24 h, 48 h.

The Transwell experiment was used to investigate cellular invasion capacity *in vitro*. KYSE-150 cells were cultured in a serum-free medium for 12–24 h to starve the cells. After matrigel hydration in a 24-well plate, serum-free medium was added to the upper chamber, and cells (5 × 10^4^) were seeded in each chamber. 10 % serum-containing medium was added to the 24-well plate as an inducer. Added the experimental samples (Control, DENV, cRGD-DENV/miR-375, cRGD-DENV-aPD-L1, cRGD-DENV-aPD-L1/miR-375) after attaching, checked cells under the chamber at the nodes of 12 h, 24 h, 48 h, terminated experiment after detecting cells, carefully wipe the matrigel and cells in the upper chamber with a cotton swab, and then in the upper and lower chambers 4 % paraformaldehyde (500 μL) was added for 30 min and finally, stained with 0.5 % crystal violet. Three fields were selected randomly under an inverted microscope to calculate the number of invading cells and the invasion rate.

### 2.8 Flow cytometry

KYSE-150 cells (5 × 10^5^) were seeded on a 6-well plate overnight, treated with PBS, DENV, cRGD-DENV/miR-375, cRGD-DENV-aPD-L1, cRGD-DENV-aPD-L1/miR-375, respectively. After 24 h, KYSE-150 cells were harvested and detected with the Annexin V-FITC Apoptosis Detection Kit according to the manufacturer's protocol, and the apoptosis ratio was analyzed by BD Accuri C6 flow cytometer and Modfit™ software (BD Biosciences, Mountain View, CA, USA).

### 2.9 RT-PCR

After treating with different treatments (PBS, DENV, cRGD-DENV/miR-375, cRGD-DENV-aPD-L1, cRGD-DENV-aPD-L1/miR-375) for 48 h, total RNA was extracted with Trizol. Then, the RNA was reverse-transcribed from the RTase according to the manufacturer's protocol. The reverse transcript primers used in this reaction system were listed in Supplementary Table 1. Subsequently, the reverse transcript cDNA (1 μL) was added to the PCR system with forward and reverse primers, respectively. Finally, RNase-free water was used to supplement the reaction system. The reaction system was amplified at 95 °C for 15 s and at 60 °C for 1 min for 40 cycles. RNU6B was used as internal control, and the relative expression of miR-375 was evaluated based on the 2^−ΔΔ*C*T^ method.

### 2.10 Western blot analysis

KYSE-150 cells were lysed with RIPA buffer comprising PMSF. Protein was mixed with 5 × loading buffer and boiled for 5 min in water at 100 °C. Equal amounts of protein (10 μg) were separated by 10 % SDS-PAGE and transferred to PVDF membranes, then blocked with 5 % BSA in TBST for 1 h at room temperature. And the membrane was incubated with monoclonal antibodies YWHAZ (1:1000) and GAPDH (1:1000) overnight. After washing, the membrane was incubated with a secondary antibody (1:2000) for 2 h at room temperature. The bands were visualized by a High-sig ECL western blotting substrate and analyzed on the Tanon 5200 Chemiluminescence imaging system (Tanon, Shanghai, China).

### 2.11 Biodistribution of DENV *in vivo*

As reported previously, the esophageal cancer-bearing mice were constructed using the heterotopic transplantation method with slight modification [23]. All procedures were approved by the Committee on the Ethics of Animal Experiments of Henan University of Science and Technology (Luoyang, China). In brief, KYSE-150 serum-free cell suspension (1 × 10^7^ cells/mL, 100 μL) was injected subcutaneously into the armpit of the right limb of Balb/c mice, and then recording weight, tumor size were recorded every three days until the experiment ended. Tumor volumes were calculated using the formula: tumor volume = (length × width^2^)/2. When the tumor volume reached 100 mm^3^, they were randomly divided into six groups with six mice in each group as follows: Group 1: Control (treated with saline), Group 2: DENV, Group 3: cRGD-DENV/miR-375, Group 4: aPD-L1, Group 5: cRGD-DENV-aPD-L1, Group 6: cRGD-DENV-aPD-L1/miR-375 (100 μL) were respectively injected every three days for seven times.

For DENV biodistribution *in vivo*, DIR-labeled DENV and cRGD-DENV (0.5 mg/mL, 100 μL) were injected into Balb/c nude mice separately by tail vein, and the fluorescence images were captured after 3 h via IVIS Lumina III In Vivo Imaging System (PerkinElmer, USA).

Balb/c mice bearing KYSE-150 tumors of 50∼100 mm^3^ were i.v. injected with saline, DENV, cRGD-DENV/miR-375, aPD-L1, cRGD-DENV-aPD-L1, cRGD-DENV-aPD-L1/miR-375. Ten minutes before sacrifice at 6 h postinjection of the conjugates, the mice were i.v. The blood vessels were stained by injecting FITC-labeled Lycopersicon esculentum lectin. The tumors were excised and sliced into sections with a thickness of 10 μm then sliced into sections with a thickness of 10 μm. Subsequently, these sections were observed using confocal microscopy. The DIR-labeled nanodrugs are red, the blood vessels are green, and the DAPI-stained nucleus is blue.

### 2.12 Tumor penetration rate

Based on the previous research methods, KYSE150 cells were used to construct esophageal tumor organoids [28], then the tumor organoids were subsequently treated individually with DiI-labeled DENV, cRGD-DENV/miR-375, cRGD-DENV-aPD-L1, and cRGD-DENV-aPD-L1/miR-375 on confocal dishes and studied with CLSM.

### 2.13 Histological analysis

With respect to biosafety, at the end of animal experiments, the mice were sacrificed, and serum was obtained at the specified times. To monitor the inflammatory response, serum TNF-α, IL-1β, and IL-6 were detected by ELISA. Simultaneously, pathological examinations of vital organs were performed to assess the biocompatibility of DENV *in vivo*. The major organs (including heart, liver, spleen, lung, and kidney) of mice in different treatment groups were collected and fixed with paraformaldehyde, followed by tissue dehydration, paraffin embedding, paraffin sectioning, and H&E staining for histological evaluation.

Paraffin-embedded tissue sections were used to detect Caspase-3 and Caspase-9 expression, as well as infiltration of lymphocytes. Tissue sections with four μm thicknesses were prepared and dewaxed with xylene I, II and III for 15 min, respectively, and hydrated in gradient ethanol (100 %, 95 %, 85 % and 75 %) for an appropriate time, then washed with PBS three times. After sections were dewaxed and rehydrated, the antigen was repaired in boiling citric acid buffer (0.01 M, pH = 6) for 15 min. Then, the universal SP kit mouse/rabbit streptavidin-biotin method detection system was used. For immunohistochemical analysis, sections were incubated at 4 °C overnight with anti-caspase 9, anti-caspase 3, anti-CD3, anti-CD4 and anti-CD8 antibodies, respectively. Labeling was identified by application of a goat anti-rabbit IgG/HRP secondary antibody at 37 °C for 30 min. After washing with PBS, the sections were incubated with 3,3′-diaminobenzidine (DAB), rinsed in water, counterstained with hematoxylin, and differentiated with hydrochloric acid and ethanol. Finally, slides were observed using a ZEISS Axio Imager system.

### 2.14 Statistical analysis

Data were presented as the mean ± SDs deviation of three technical replicates. Statistical analysis was performed using the two-tailed *t*-test or one-way analysis of variance (ANOVA) to assess statistical significance between groups. A *P* value < 0.05 was considered statistically significant (∗*P* < 0.05; ∗∗*P* < 0.01; ∗∗∗*P* < 0.001).

## 3 Results

### 3.1 Preparation and characterization of DENV-based delivery system

In this research, DENV was isolated from the supernatant of *D. salina* by multistep differential centrifugation, and the BCA protein assay was employed to ascertain the protein concentration of the DENV. According to the results, about 100 μg DENV (approximately 1.2 × 10^11^ particles) could be prepared from 100 mL culture supernatant, and multiple rounds of separation can obtain the concentration required for the experiment.

**Fig. 2:**
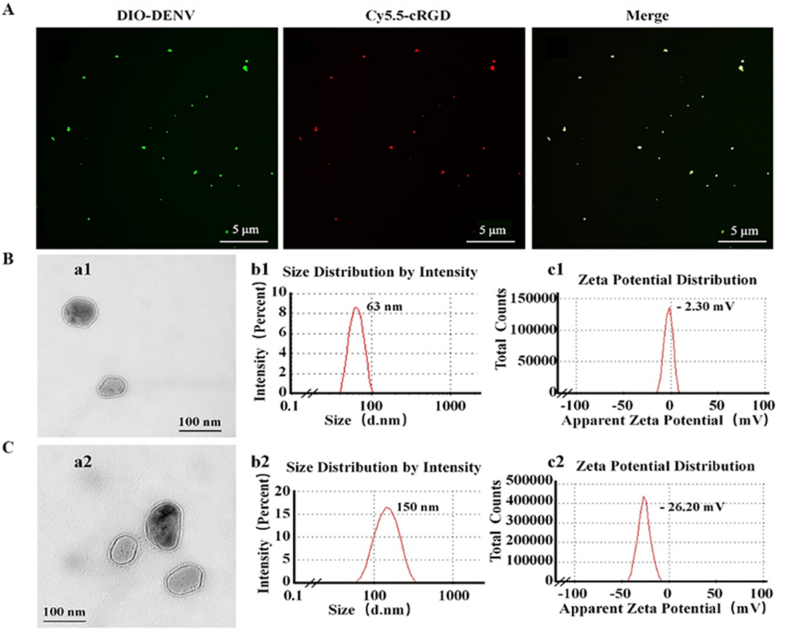
Characterization of DENV and cRGD-DENV-aPD-L1/miR-375. (A) Fluorescent overlay of DENV loaded with cRGD peptide (Scale bars 5 μm). (B) The morphology (a1), size (b1), and Zeta potential (c1) of DENV. (C) The morphology (a2), size (b2), and Zeta potential (c2) of cRGD-DENV-aPD-L1/miR-375 (Scale bars 100 nm).

The cRGD peptide was modified on the DENV by chemical coupling to achieve tumor targeting *in vivo*. Firstly, DENV was labeled with DiO, cRGD was labeled with Cy5.5-NHS, and conjugating consequences were recorded under a confocal laser scanning microscope (CLSM) (Fig. 2A). The result of fluorescence co-localization indicated that green fluorescence overlaps with red fluorescence, and a yellow fluorescence bright spot appeared. This phenomenon suggests the successful coupling of DENV and cRGD peptide.

Then, TEM was used to identify the morphology of engineered DENV, as shown in Fig. 2B and C. DENV presented a typical “disc” shape with an average diameter of about 60 nm (Fig. 2B–a1), which was analogous to the reported exosome size in mammalian cells [29]. Then, DENV size was analyzed using a dynamic light scattering particle size analyzer (DLS), and the particle size distribution of DENV in aqueous solution was mainly concentrated at 63 nm (Fig. 2B–b1), which was roughly consistent with the results observed under TEM. Moreover, the size of cRGD-DENV-aPD-L1/miR-375 was increased to around 150 nm (Fig. 2C–a2 and 2C-b2), slightly larger than DENV. Next, we detected the Zeta potential value. As shown in Fig. 2B and c1, the Zeta potential of DENV was about −2.30 mV. However, after loading miR-375 and aPD-L1, the Zeta potential was approximately −26.2 mV (Fig. 2C–c2), which revealed that negatively charged miR-375 increased the negative charge. It was attributed to the negative charge to surface components of the engineered DENV (e.g., exposed anionic residues from cRGD or aPD-L1 conjugation). Furthermore, we test its stability, the morphology and Zeta potential remained basically unchanged after the DENV were placed in a −4 °C for one month. These results are consistent with the characterization of typical exosomes, confirming the successful isolation of DENV and formulation preparation of cRGD-DENV-aPD-L1/miR-375.

### 3.2 Cell uptake of DENV-based delivery system

**Fig. 3:**
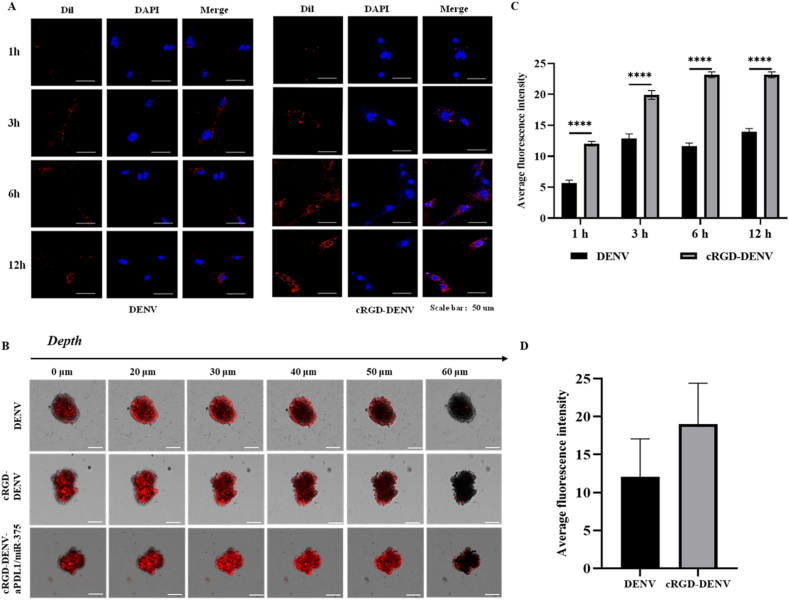
Cell uptake of DENV-based delivery system. (A) Observation of DIO-labeled DENV entering esophageal cancer cells under CLSM at different time points (Scale bars 50 μm). (B) The infiltration degree of exosomes in tumor spheres (Scale bars 50 μm). (C) Quantitative analysis of the fluorescence of exosomes taken up by cells at different times. (D) Analysis of the average fluorescence intensity of 3D tumor spheres in different groups.

To explore the internalization kinetics of DENV modified with cRGD polypeptide within KYSE-150 cells, confocal laser-scanning microscopy (CLSM) fluorescence images of KYSE-150 cells were captured at 1 h, 3 h, 6 h, and 12 h post-incubation with DiI-labeled DENV (DiI-DENV, 0.5 mg/mL). Fig. 3A shows the series of images of DiI-DENV entering esophageal cancer cells. Analysis of the images showed that the intensity of red fluorescence in KYSE-150 cells increased to the 6 h time point, and the trend remained unchanged at 12 h. The decrease of DiI fluorescence may result from the processing of internalized DENV by KYSE-150 cells and/or fluorescence quenching of the DiI molecules. Remarkably, cRGD-DENV (right) was internalized more by KYSE-150 cells in contrast to DENV (left), which showed that cRGD modification can significantly enhance the internalization of DENV in KYSE-150 cells and cRGD-DENV is concentrated around the nucleus, further indicating that cRGD-DENV exhibits better targeting properties *in vivo*.

In order to simulate tumors *in vivo* and further study the penetration of engineered nanoparticles into tumors, we constructed esophageal tumorspheres. When the diameter of the spheres reached 100–150 nm, the DiI-labeled exosomes were incubated with the tumor spheres for 4 h, and the penetration ability of exosomes into tumors was investigated by confocal microscope layer scanning. The presence of fluorescence in the center of the sphere indicates that exosomes penetrate the sphere's depth. As shown in Fig. 3B, the central fluorescence signal of tumor spheres in the cRGD-DENV group and the CRGD-DENV-APDL1/miR-375 group was significantly enhanced compared with that in the DENV group. When the layer scanning reached 60 μm, the fluorescence signal of tumor spheres in the CRGD-DENV group and CRGD-DENV-APDL1/miR-375 group was still higher than that in the DENV group, indicating that cRGD could promote the penetration of DENV into tumor spheres.

### 3.3 Release of aPD-L1 in vitro

**Fig. 4:**
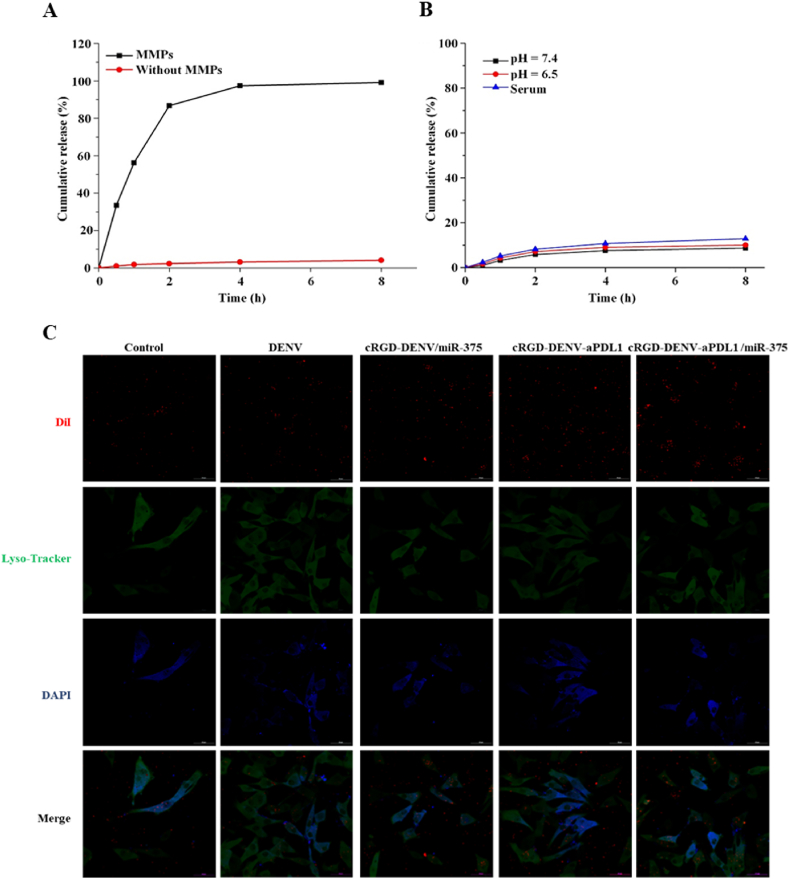
Assay of aPD-L1 release in PBS and serum and co-localization of engineered DENV and lysosome tracker red after 6 h of culture with KYSE-150 cells. (A) Release of aPD-L1 from cRGD-DENV-aPD-L1/miR-375 was determined in the medium of PBS containing MMPs or not. (B) *In vitro* release of aPD-L1 from cRGD-DENV-aPD-L1/miR-375 in PBS (pH = 7.4 and pH = 6.5) and serum. (C) The DiI-labeled nanodrugs were shown in Red, the lysosome tracker red was shown in Green, and the DAPI-stained nucleus was shown in blue (Scale bars 50 μm).

To achieve TME-triggered rapid release of aPD-L1 in the vicinity of tumor tissues, Gly-PLGLAG-Cys, a substrate peptide exquisitely sensitive to MMPs, which are highly expressed in a broad spectrum of tumor types [30], was selected as the linker between DENV and aPD-L1. Firstly, we studied the release of aPD-L1 from cRGD-DENV-aPD-L1/miR-375. As shown in Fig. 4A, aPD-L1 conjugated on the surface of cRGD-DENV-aPD-L1/miR-375 was nearly completely released within 8 h in PBS containing MMPs. In contrast, only a few aPD-L1 were released in the medium of PBS without MMPs. In addition, there were few effects in PBS (pH 7.4 and 6.5) and serum on the release of aPD-L1 from cRGD-DENV-aPD-L1/miR-375 *in vitro* (Fig. 4B). These results indicated that cRGD-DENV-aPD-L1/miR-375 was highly sensitive to MMPs, and rapidly released aPD-L1 in the presence of MMPs.

Then, to distinguish whether engineered DENV was transported to lysosomes after internalization, the KYSE-150 cells were stained with a lysosome marker (LysoTracker Green), and engineered DENV was labeled with DiI (Red). As displayed in Fig. 4C, only a few signals of the internalized engineered DENV were co-localized with lysosomes. While most green fluorescent signals are distributed around the cytoplasm. This finding suggests that only a small amount of the engineered DENV in each group enters lysosomes, which stands in contrast to most nanoformulations that undergo lysosomal degradation. The probable reason may be the presence of specific molecules expressed on DENV, resulting in lysosome escape of engineered DENV.

### 3.4 Inhibition of cell proliferation, migration and invasion

**Fig. 5:**
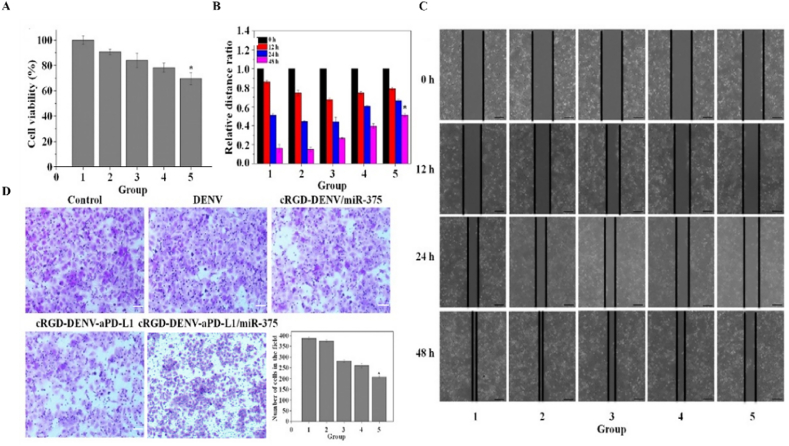
The effect of engineered DENV on proliferation, migration and invasion of esophageal cancer cells. (A) The cytotoxicity of engineered DENV (Group1: Control, Group2: DENV, Group3: cRGD-DENV/miR-375, Group4: cRGD-DENV-aPD-L1, Group5: cRGD-DENV-aPD-L1/miR-375). (B) Quantitative analysis based on wound-healing results in (C). (C) Cell wound healing experiment treated with different samples in KYSE-150 cells at 0 h, 12 h, 24 h and 48 h. (D) Transwell experiment treated with different samples in KYSE-150 cells at 48 h (∗*p* < 0.05, when compared with control).

Then, cRGD-DENV loaded with miR-375 and aPD-L1 was employed to investigate the impacts on proliferation, migration and invasion of esophageal cancer cells *in vitro*. As shown in Fig. 5A, the cRGD-DENV-aPD-L1/miR-375 group exhibited a significantly more significant inhibitory effect compared to the other treatment groups alone, indicating that the combination of aPD-L1 and miR-375 exerted the optimal suppressive effect on KYSE-150 cells. Moreover, we used the wound-healing and Transwell experiments to study the migration and invasion of the delivery system on KYSE-150 cells. In Fig. 5B and C, after 72 h, the wound-like gaps in the untreated and DENV-treated cells had healed almost completely, while the cRGD-DENV-aPD-L1/miR-375 group remained a large gap. As shown in Fig. 5D, cell invasion inhibition was similar to cell migration in different treatment groups. Taken together, the cRGD-DENV-aPD-L1/miR-375 group manifested a manifested inhibitory impact on the migration and invasion capabilities of KYSE-150 cells.

### 3.5 Flow cytometry assay and gene expression monitoring

**Fig. 6:**
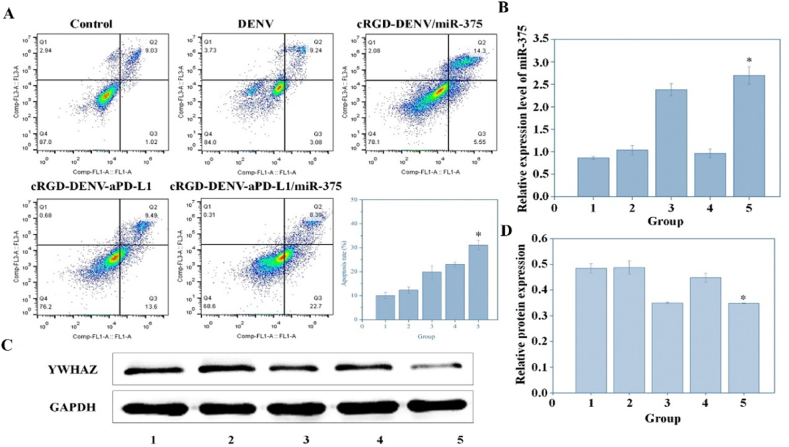
Flow cytometry, PCR and Western blot analysis of KYSE-150 cells after treating with PBS, DENV, cRGD-DENV/miR-375, cRGD-DENV-aPD-L1 and cRGD-DENV-aPD-L1/miR-375. (A) Cell apoptosis of KYSE-150 cells treated with different samples within 48 h. (B) The expression of miR-375 in KYSE-150 cells. (C) Western blot analysis of YWHAZ expression in KYSE-150 cells. (D) Quantitative analysis based on YWHAZ expression in (C). (Group1: Control, Group2: DENV, Group3: cRGD-DENV/miR-375, Group4: cRGD-DENV-aPD-L1, Group5: cRGD-DENV-aPD-L1/miR-375. ∗*p* < 0.05, when compared with control).

Next, we investigated the effect of the engineered DENV on KYSE-150 cells. Flow cytometry detected the cellular apoptosis rate after treatment with different formulations for 48 h. As shown in Fig. 6A, the early apoptosis rate of KYSE-150 cells with different treatments was about 1.02 % (untreated), 3.08 % (DENV), 5.5 % (cRGD-DENV/miR-375), 13.6 % (cRGD-DENV-aPD-L1) and 22.7 % (cRGD-DENV-aPD-L1/miR-375), respectively. The cRGD-DENV-aPD-L1/miR-375 exhibited a higher early apoptotic induction rate compared with the control group and DENV group. Moreover, the total apoptosis rate, including early and late apoptosis at five different treatments, was about 10.5 %, 12.32 %, 19.85 %, 23.09 % and 31.09 %, respectively. It was demonstrated that the combination of aPD-L1 and miR-375 had a significant statistical difference in apoptosis induction compared with control, DENV group and single drug groups (*p* < 0.05).

Research has shown that miR-375 overexpression may impact cell proliferation, migration and invasion by facilitating cell apoptosis [31]. To investigate the possible mechanism of miR-375-mediated inhibition of cell proliferation and migration, firstly, we quantified the expression levels of miR-375 in five different treatments. Quantitative PCR results (Fig. 6B) showed that miR-375 expression levels in the cRGD-DENV/miR-375 and cRGD-DENV-aPD-L1/miR-375 groups were significantly increased compared with other groups. Next, YWHAZ, a predicted and identified target gene of miR-375, plays essential roles in regulating cell apoptosis, invasion and migration [32], which was selected to investigate the effect of engineered DENV on esophageal cancer cells. As shown in Fig. 6C and D, the YWHAZ protein expression levels in the cRGD-DENV/miR-375 group and the cRGD-DENV-aPD-L1/miR-375 group were significantly decreased than the control group, and the cRGD-DENV-aPD-L1/miR-375 group significantly inhibited YWHAZ expression. Therefore, cRGD-DENV-aPD-L1/miR-375 successfully delivered miR-375 to esophageal cancer cells to exert an inhibitory effect, affecting proliferation, migration and invasion of esophageal cancer cells by regulating YWHAZ.

### 3.6 In vivo targeting and anti-tumor effects of engineered DENV

**Fig. 7:**
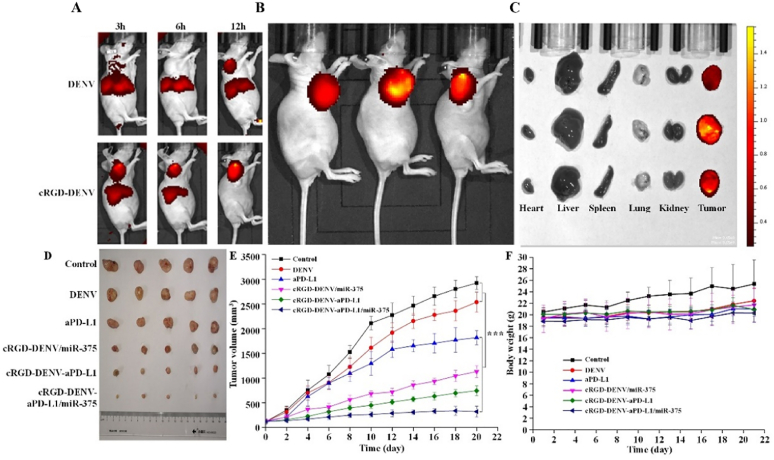
Anti-tumor evaluation *in vivo*. (A) The NIRF imaging of mice at different times after administration of DENV and cRGD-DENV. (B) The NIRF imaging of mice at 12 h after administration cRGD-DENV. (C) The NIRF imaging of main organs at 12 h after administration cRGD-DENV. (D–E) Tumor sizes and volumes of nude mice after being treated with saline (Control), DENV, cRGD-DENV/miR-375, aPD-L1, cRGD-DENV-aPD-L1, cRGD-DENV-aPD-L1/miR-375 by tail vein injection. (F) Body weight changes of each group during treatment. (∗∗∗*p* < 0.001 when compared with control).

cRGD-DENV mediated co-delivery of miR-375 and aPD-L1 to the KYSE-150 cells had shown an ideal delivery effect *in vitro*. Subsequently, their delivery efficiency *in vivo* was evaluated by IVIS Lumina III *In Vivo* Imaging System (PerkinElmer, USA). Firstly, we investigated the targeting of the DIR-labeled DENV and cRGD-DENV *in vivo*. After tail vein injection of DENV and cRGD-DENV, the biodistribution of injected DENV was monitored at different time points (Fig. 7A). At the earliest time point after injection (3 h), DENV and cRGD-DENV were rapidly distributed all over the body, with the difference that a large proportion of cRGD-DENV was distributed in the tumor at 12 h, which was indicative of the fast accumulation within the tumor area. Whereas DENV was located mainly in the liver, suggesting that DENV uptake and retention took place primarily in the liver and other metabolic organs, with little accumulation in the tumor. However, as time went on, striking differences were found between DENV and cRGD-DENV groups. A relatively intense fluorescence signal exclusively in the tumor area was detected after 6 h of injection of cRGD-DENV, while other parts of the body gradually decreased. Representative results are shown in Fig. 7A. Moreover, the relatively intense fluorescence signal could still be detected in the liver after 12 h of injection of DENV. In contrast, almost no signal was detected at the liver in the cRGD-DENV group, and the fluorescence signal could only be detected in tumor sites (Fig. 7A). Fig. 7B and C showed the representative images in the cRGD-DENV group, indicating a rapid blood clearance by the reticuloendothelial system. These data suggested that cRGD-DENV could be an effective drug delivery carrier targeting the integration-expressing tumor.

Furthermore, the anti-tumor effect of engineered DENV *in vivo* has been investigated. Compared with the control group, Balb/c mice injected with cRGD-DENV-aPD-L1 and cRGD-DENV-aPD-L1/miR-375 had remarkable tumor inhibition effects. Notably, the cRGD-DENV-aPD-L1/miR-375 group significantly inhibited tumor growth and decreased tumor volume (Fig. 7D and E). When cRGD-DENV-aPD-L1/miR-375 arrived at the tumor site, the matrix metalloproteinase overexpressed in TME could cleave Gly-PLGLAG-Cys, and aPD-L1 was released and interacted with PD-L1 receptors on tumor cells, which can enhance the anti-tumor effect of the immune system *in vivo*. Subsequently, miR-375 was delivered to tumor cells and then played a role in inhibiting tumor growth. Additionally, during the treatment, no obvious weight loss was observed in each group (Fig. 7F), suggesting the safety of this targeted delivery system based on DENV. These results further demonstrated the successful delivery of aPD-L1 *in vivo* and the favorable outcome of the combination of immune and gene therapy.

### 3.7 Histological analysis

Next, we evaluated the penetration of the engineered DENV in tumors. As presented in Supplementary Fig. 1, red fluorescent bright spots appear at the tumor sites, which shows that the DENV delivery system could effectively reach the tumor sites. Moreover, DIR-labeled DENV (red) in cRGD-DENV/miR-375, cRGD-DENV-aPD-L1 and cRGD-DENV-aPD-L1/miR-375 groups were primarily retained in the periphery of the blood vessels (green) than the DENV group, demonstrating that cRGD peptide greatly facilitates the tumor penetration of engineered DENV, which further indicates cRGD peptide has better-targeting properties to esophagus cancer *in vivo*.

**Fig. 8:**
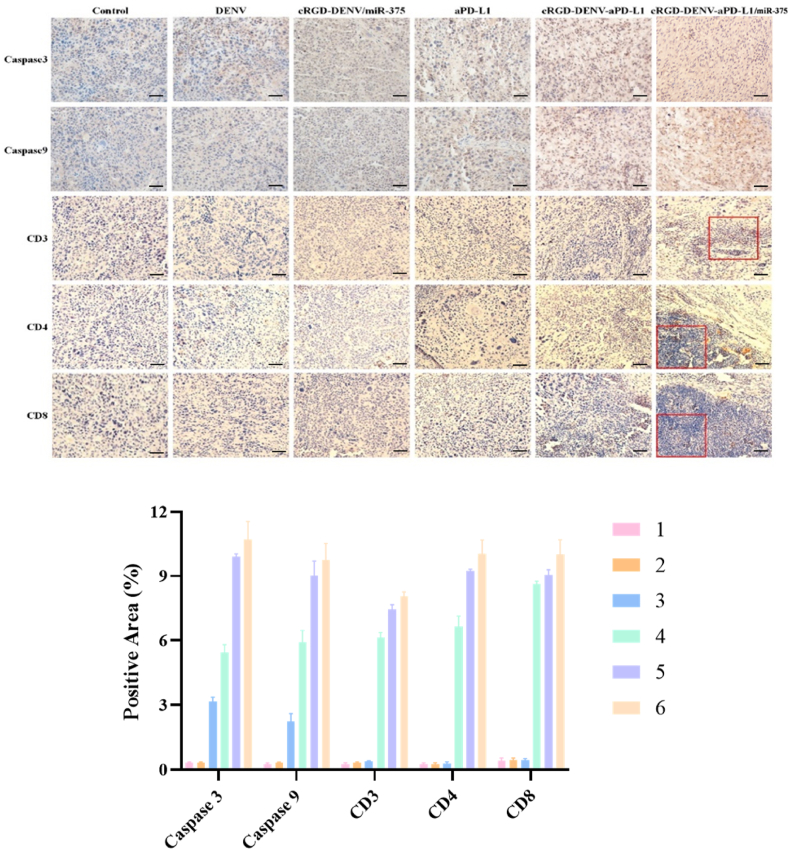
Immunohistochemical analyses of Caspase 3, Caspase 9, CD3, CD4 and CD8 for tumor tissues (Scale bars 50 μm). (Group1: Control, Group2: DENV, Group3: cRGD-DENV/miR-375, Group4: aPD-L1, Group5: cRGD-DENV-aPD-L1, Group6: cRGD-DENV-aPD-L1/miR-375).

Caspase 3 and caspase 9 are commonly used indicators in the apoptosis of various cells. Therefore, at the end of the investigation, the mice were sacrificed and tumor tissue sections were cut and subjected to apoptosis detection. As shown in Fig. 8, the expressions of caspase 3 and caspase 9 in the cRGD-DENV-aPD-L1/miR-375 group were higher than other groups, indicating that the successful delivery of aPD-L1 *in vivo* and had notable anti-tumor effects in tumor sites. Studies have shown that treating with DC-TEX (tumor-derived exosome-pulsed dendritic cells) could increase CD8^+^ T cells and suppress tumor growth in orthotopic HCC mice [33,34], while the inhibition of tumor growth caused by anti-SEMA4 blocking therapy in colorectal cancer tissue depended on the infiltrating CD8^+^ T cells in tumor tissue [35]. Subsequently, we assessed the infiltration of lymphocytes by detecting the expression of CD3, CD4 and CD8 to determine how immune cell populations were affected by aPD-L1. As we can see in Fig. 8, the groups of aPD-L1, cRGD-DENV-aPD-L1 and cRGD-DENV-aPD-L1/miR-375 had lymphocyte infiltration compared with the control group, and the cRGD-DENV-aPD-L1/miR-375 group had the most lymphocyte infiltration, which indicated cRGD-DENV-aPD-L1/miR-375 could recruit more lymphocytes to combat tumor cells, thereby inhibiting tumor growth.

### 3.8 In vivo safety evaluation

**Fig. 9:**
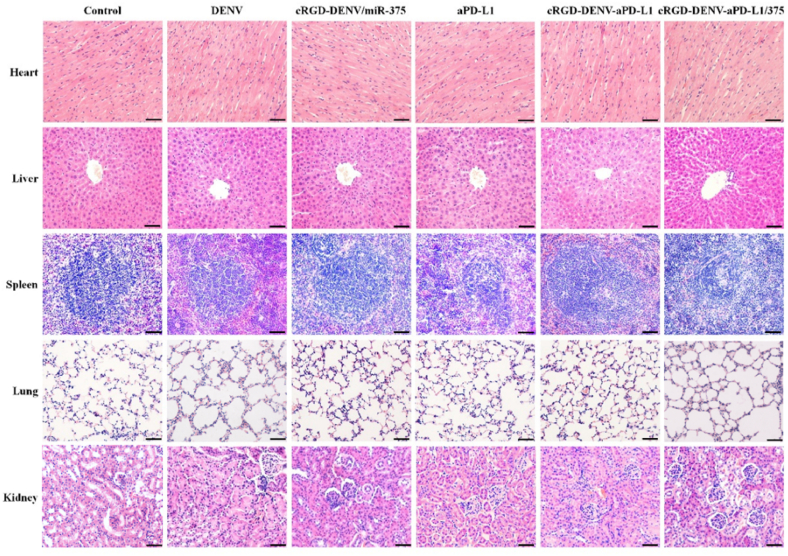
HE staining of significant organs after treated with saline (Control), DENV, cRGD-DENV/miR-375, aPD-L1, cRGD-DENV-aPD-L1, cRGD-DENV-aPD-L1/miR-375 (Scale bars 50 μm).

In addition to treatment efficacy, toxicity is another critical consideration of an excellent delivery system for clinical application. For safety purposes, we evaluated the systemic toxicity of the different formulations in healthy Balb/c mice after intravenous injection. Compared with the control group, no deaths and severe body weight loss were observed for the test groups. We further investigated the potential pathological lesions induced by the formulations on significant organs. Blood biochemistry and hematology analysis were carried out to reveal any potential toxic effect of DENV on the treated mice. Different biochemistry parameters were tested, including the liver function markers, such as ALT, AST, and the kidney function markers, such as CRE, BUN. As can be seen in Supplementary Table 2, all the above index levels remained the same as the untreated group, indicating that cRGD-DENV-aPD-L1/miR-375 had no apparent hepatic or renal toxicity within the dosage regimen. White blood cell (WBC) counts were examined for the hematological assessment. All the above parameters of the cRGD-DENV-aPD-L1/miR-375 group indicated no significant difference compared with the control group (Supplementary Table 2). Furthermore, as shown in Fig. 9, primary tissues (including heart, liver, spleen, lung and kidney) had no noticeable histopathological abnormalities or lesions in the cRGD-DENV-aPD-L1/miR-375 group. We further quantified the serum levels of IL-6, IL-1β and TNF-α, and no significance was observed among these groups (Supplementary Fig. 2), which suggested that there was no evidence of inflammatory response caused by cRGD-DENV-aPD-L1/miR-375. These results showed that multiple dosages of cRGD-DENV-aPD-L1/miR-375 did not cause acute toxicity to mice's hematological system and major organs.

## 4 Discussion

As a nanoscale membrane vehicle, exosomes are believed to be promising carriers of their unique properties, including low immunogenicity, biodegradability, low toxicity and effective protection for cargo [36]. Recently, exosome-like nanovesicles from microbial and plants to mammals have been investigated. However, a crucial disadvantage is that clinical applications require many exosome-like nanovesicles. Large-scale cell culture is indispensable [37]. However, this approach demands substantial production costs and a significant amount of time. Moreover, mammalian cell cultures necessitate the utilization of animal-derived components, like fetal bovine serum (FBS) [38]. Owing to safety apprehensions, the use of such components is typically restricted in the drug approval process. Additionally, the production of exosomes derived from milk and morning urine is relatively high (approximately 33 × 10^16^ particles and 2.4 × 10^12^ particles could be generated from 1L of bovine milk and 300 mL of one adult's morning urine [39]. Nevertheless, exosomes derived from milk also have the above-mentioned safety problems, and acquiring morning urine requires good patient compliance. It is noteworthy that the *D. salina* used in this study has a short growth cycle (about two weeks), and exosome-like nanovesicles with high yield and purity can be isolated at any time (approximately 1.2 × 10^11^ DENV could be generated from 100 mL *D. salina* culture supernatant), which greatly reduces production costs and time. While natural exosomes benefit from natural cell-recognition properties, engineered DENV exhibit superior functional tunability, making them more suitable for tailored therapeutic applications like targeted cancer therapy.

Furthermore, tumor targeting is a key performance criterion for nanocarriers, and exosomes stand out as exceptional natural carriers, primarily attributed to their inherently low toxicity and minimal immunogenicity. This unique combination of properties makes them highly suitable for a wide range of applications. However, the intravenous delivery of exosomes into target sites remains a challenge because of the suboptimal targeting performance exhibited by unmodified exosomes [27]. The solid tumors have a dense extracellular matrix (ECM), abnormal vascular structures and high interstitial pressure, which makes it difficult for drugs to penetrate deep into the tumor, and this is a key obstacle leading to treatment failure [40]. To achieve tumor targeting *in vivo*, targeting ligands (such as antibodies and polypeptides) were conjugated to the exosomal surface to enhance specific interactions between exosomes and target cells [29]. The RGD peptide binds tightly with α/β integrin, which is highly expressed on tumor cells and promotes drug penetration into blood vessels or tissues. As previously reported, αvβ3 is highly expressed in esophageal cancer tissues and cell lines [41]. This finding indicates that esophageal cancer may serve as a potential target for RGD-decorated exosomes. In this study, we successfully developed and characterized cRGD-modified exosome-like nanovesicles derived from Dunaliella salina (cRGD-DENV). As expected, cRGD-DENV showed enhanced cellular uptake compared with unmodified DENV *in vitro* (Fig. 3), and cRGD-DENV focused on the tumor sites distinctly *in vivo* after intravenous injection (Fig. 6A–C). Interestingly, the cRGD-DENV can better accumulate in the tumor site and is less uptaken by macrophages in the liver, which was different from other reports that most intravenous EVs are absorbed in the liver [42]. Notably, the enhanced tumor targeting of DENV at 12 h *in vivo* (Fig. 7B) compared to *in vitro* (Fig. 3) likely stems from physiological factors: *in vivo*, the tumor microenvironment (e.g., leaky vasculature via the EPR effect) and active homing via cRGD-integrin interactions facilitate prolonged retention and accumulation. In contrast, *in vitro* assays reflect acute internalization kinetics without cRGD modification, such as systemic or microenvironmental support. In conclusion, these consequences showed that cRGD-DENV had excellent targeting and could quickly reach the tumor sites. This further indicated that exosome-like nanovesicles modified by cRGD could be an effective drug delivery vehicle for targeting α_v_β_3_-expressing tumors.

Over the last decade, nanomedicine integrating immunotherapy and gene therapy into a nano-platform has been widely proven to be an essential paradigm for cancer treatment, and miRNAs have been shown to exert a functional impact on numerous cancer markers [43]. Studies have found that the expression of miR-375 is notably low in ESCC, and its downregulation has been reported to be a promising prognostic biomarker for ESCC patients [44,45]. Therefore, we developed a targeted delivery system based on DENV to simultaneously deliver miR-375 and aPD-L1 into KYSE 150 cells to increase the effectiveness of the cancer treatment. We found that engineered DENV can inhibit the proliferation, migration, and invasion of KYSE 150 cells. More importantly, cRGD-DENV-aPD-L1/miR-375 can significantly induce apoptosis in KYSE 150 cells, similar to other researchers. Our *in vivo* studies demonstrated monotherapy of engineered DENV had a particular tumor inhibition effect, while cRGD-DENV-aPD-L1/miR-375 could result in significant tumor regression. These results illustrated that aPD-L1 and miR-375 were delivered successfully and the synergistic treatment had achieved excellent therapeutic effects, further indicating that this nanosystem could achieve an efficient combination of gene therapy and immunotherapy. This strategy combines the penetration property of the small-sized vector, the targeting property of cRGD, and the immunomodulatory effect, which can exert a synergistic therapeutic effect. On one hand, precise targeted delivery can make aPD-L1 and miR-375 more effectively act at the tumor site, improving the efficiency of immunomodulation; on the other hand, by relieving immune suppression and activating T cells, it can enhance the recognition and killing of tumor cells by the body, overcoming the limitations of single treatment methods and improving the therapeutic effect. To sum up, the current study provides strong evidence that exosome-like nanovesicles derived from *D. salina* are novel therapeutic bioactive materials with broad potential for biomedical applications.

## 5 Conclusions

In this study, we successfully developed a combined strategy of exosome-like nanovesicle-based co-delivery of miR-375 and aPD-L1 to enhance tumor-targeted therapy efficiency. More importantly, the exosome-like nanovesicles can be obtained in a high yield with less cost and time. Moreover, the exosome-like nanovesicle from *D. salina* with excellent biocompatibility was more likely to accumulate at tumor sites than other nano-delivery systems. In summary, the DENV-based delivery system combined with gene therapy and immunotherapy is suitable for large-scale production, which may contribute to the establishment of a synergistic treatment with the potential to be translated into clinics shortly.

## CRediT authorship contribution statement

**Zhaoyi Wei:** Writing – original draft, Software, Data curation. **Mengxi Zhu:** Validation, Formal analysis. **Shan Li:** Software. **Junling An:** Investigation, Conceptualization. **Yiwen Liu:** Investigation. **Shuying Feng:** Methodology. **Tingting Yang:** Formal analysis. **Shegan Gao:** Project administration. **Gaofeng Liang:** Writing – review & editing, Visualization, Project administration, Methodology, Funding acquisition, Data curation, Conceptualization.

## Availability of data and materials

All datasets used and analyzed during the current study are available from the corresponding author on reasonable request.

## Ethics approval and consent to participate

The Ethical Committee of Henan University of Science & Technology provided approval to conduct this study. The ethical conduct of all animal experimentation adhered strictly to the principles delineated in the Guide for the Care and Use of Laboratory Animals by International Committees.

## Funding

This work was financially supported by the National Key Research and Development Program of China (2022YFE132800), the Joint Fund of Henan Province Science and Technology R&D Program(225200810020), the Key R&D project of Henan Province (221111310600) and Special Foundation for Basic Research Program of Higher Education Institutions of Henan Province (22ZX005) to Liang Gaofeng.

## Declaration of competing interest

The authors declare that they have no known competing financial interests or personal relationships that could have appeared to influence the work reported in this paper.
