# Supplementary material for: Exosome-like nanovesicles from Dunaliella salina efficient sequential Co-delivery of anti-PDL1 and miR-375 for enhancing gene/immune therapy
Source: Noncoding RNA Res. 2025 Sep 1;14:191–203. doi: 10.1016/j.ncrna.2025.08.007 (PMC12504820; doi:10.1016/j.ncrna.2025.08.007)
Supplement: Multimedia component 1 [file mmc1.pdf]

GENEWIZ®
Solid science. Superior service.

Cell Line Authentication Report

GENEWIZ,Inc. Beijing

30 Science Park Road

Zhong-Guan-Cun Life Science Park

Changping District, 102206

Beijing, China

Tel: 400-8100-669

Fax: 010-59458058

Email: Genomics.China@genewiz.com.cn

www.genewiz.com.cn

GENEWIZ,Inc. Beijing
30 Life Science Park Road
Changping District, 102206
Beijing, China
Tel: 400-8100-669
Fax: 010-59458058
www.genewiz.com.cn

GENEWIZ®
Solid science. Superior service.

Cell Line Authentication Report

Customer: Kong JinYu

Institution: First affiliated hospital of henan university of science and technology

Quotation Number: BJ11202

Completion Date：08/10/2023

1. **Sample ID: KYSE150**
2. **Original Material: gDNA**
3. **Methods:**

1).Genomic DNA was extracted from the cell pellets provided by the customer.

2).Samples, together with positive and negative control were amplified using GenePrint 10 System(Promega).

3). Amplified products were processed using the ABI3730xl Genetic Analyzer.

4).Data were analyzed using GeneMapper4.0 software and then compared with the ATCC, DSMZ or JCRB databases for reference matching.

4. **Results:**

1) 10 Loci STR Profile：

Genetic Site	Customer sample	
(Locus)	KYSE150	
Amelogenin	X	
CSF1PO	12	13
D13S317	8	11
D16S539	9	11
D5S818	12	13
D7S820	10	11
THO1	7	9
TPOX	8	
vWA	16	17
D21S11	30	31

GENEWIZ

Solid science. Superior service.

GENEWIZ,Inc. Beijing
30 Life Science Park Road
Changping District, 102206
Beijing, China
Tel: 400-8100-669
Fax: 010-59458058
www.genewiz.com.cn

Addendum: Comparative output from the ATCC STR Profile database

Result of STR matching analysis by your data.

- DSMZ Profile Database -

A graphical presentation is shown at the bottom of this page.

EV	Cell No.	Cell name	Locus names									Figures
			D5S818	D13S317	D7S820	D16S539	VWA	TH01	AM	TPOX	CSF1PO	
	Query (Your Cell)		12, 13	8, 11	10, 11	9, 11	16, 17	7, 9	X	8	12, 13	
1.06(36/34)	375	KYSE-150	12,13	8,11	10,11	9,11	16,17	7,9	X,X	8,8	12,13	–
0.94(32/34)	HTB-178	NCI-H596 [H596]	11,13	11,11	11,11	11,11	16,16	7,9.3	X,X	8,8	12,13	–
0.94(32/34)	IFO50309	SKG-II	12,12	8,8	11,11	11,11	16,18	7,9	X,X	8,9	12,12	–
0.94(32/34)	IFO50322	NJG	13,13	11,11	10,10	12,12	16,16	9,9	X,X	8,8	13,13	–
0.94(32/34)	JCRB0175	SNG-II	12,12	8,8	11,11	11,11	16,18	9,9	X,X	8,9	12,12	–
0.94(32/34)	RCB0685	SKG-II-SF	12,12	8,8	11,11	11,11	16,18	9,9	X,X	8,9	12,12	–
0.94(32/34)	RCB0808	MMAc	12,12	8,12	11,11	9,12	17,17	9,9	X,X	8,8	12,12	–
0.94(32/34)	RCB1200	"MMAc · SF"	12,12	8,12	11,11	9,12	17,17	9,9	X,X	8,8	12,12	–
0.91(32/35)	JCRB0185	HCC-48	12,12	11,11	11,11	11,13	16,17,18	7,9	X,X	8,11	12,13	–
0.88(30/34)	649	HCEC-H9C1	11,11	8,11	10,11	11,11	14,17	9,9	X,X	8,8	12,12	–
0.88(30/34)	CRL-2258	HL-60/MX1	12,12	8,11	11,12	11,11	16,16	7,8	X,X	8,8	13,14	–
0.88(30/34)	IFO50358	KNS-62	9,9	11,11	11,11	9,11	17,17	7,9	X,X	8,8	9,12	–

2) Electrophoretogram

GENEWIZ,Inc. Beijing
30 Life Science Park Road
Changping District, 102206
Beijing, China
Tel: 400-8100-669
Fax: 010-59458058
www.genewiz.com.cn

Applied Biosystems
GeneMapper 4.0

BJ11199

Sample File	Sample Name	Panel	OS	SQ
KYSE150.fsa	KYSE150	GenePrint_10_v1.1		

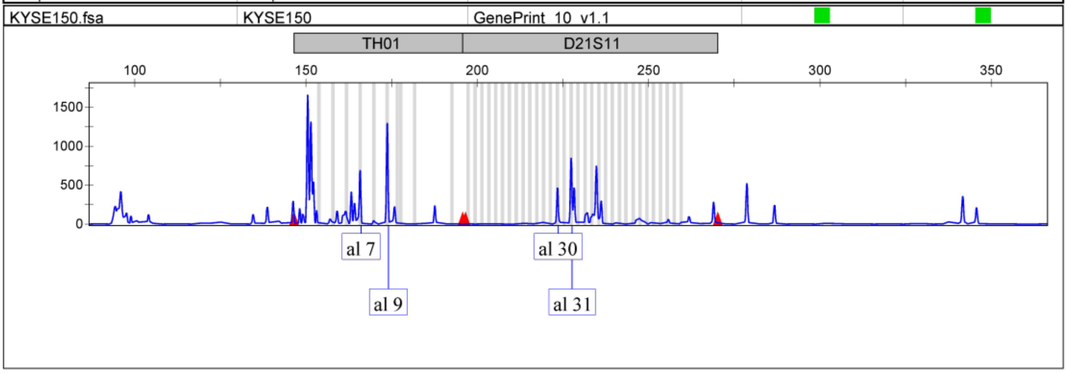

KYSE150.fsa	KYSE150	GenePrint_10_v1.1		

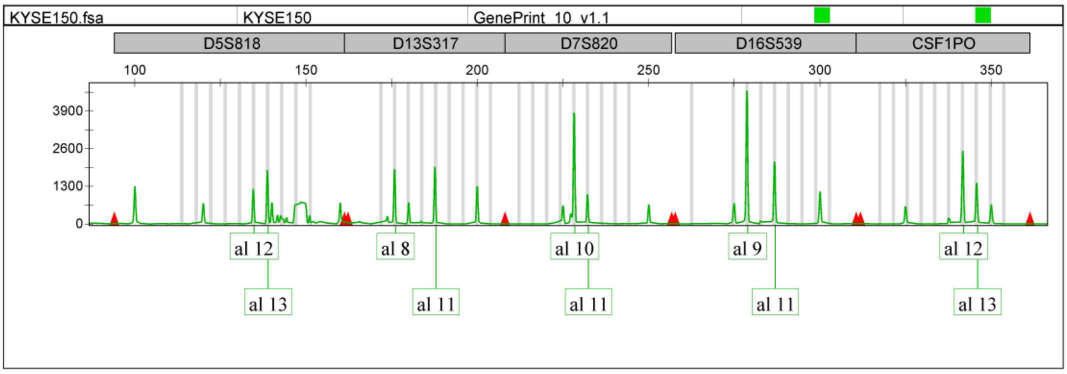

KYSE150.fsa	KYSE150	GenePrint_10_v1.1		

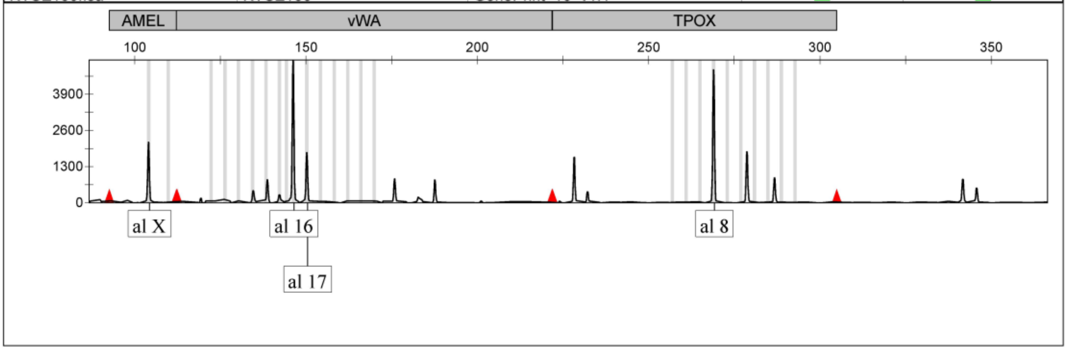

Note： Raw data in appendix
