# Supplementary material for: Exosome-like nanovesicles from Dunaliella salina efficient sequential Co-delivery of anti-PDL1 and miR-375 for enhancing gene/immune therapy
Source: Noncoding RNA Res. 2025 Sep 1;14:191–203. doi: 10.1016/j.ncrna.2025.08.007 (PMC12504820; doi:10.1016/j.ncrna.2025.08.007)
Supplement: Multimedia component 2 [file mmc2.docx]

**Supplementary information**

**Exosome-like nanovesicles from Dunaliella salina Efficient Sequential Co-Delivery of anti-PDL1 and miR-375 for enhancing gene/immune therapy**


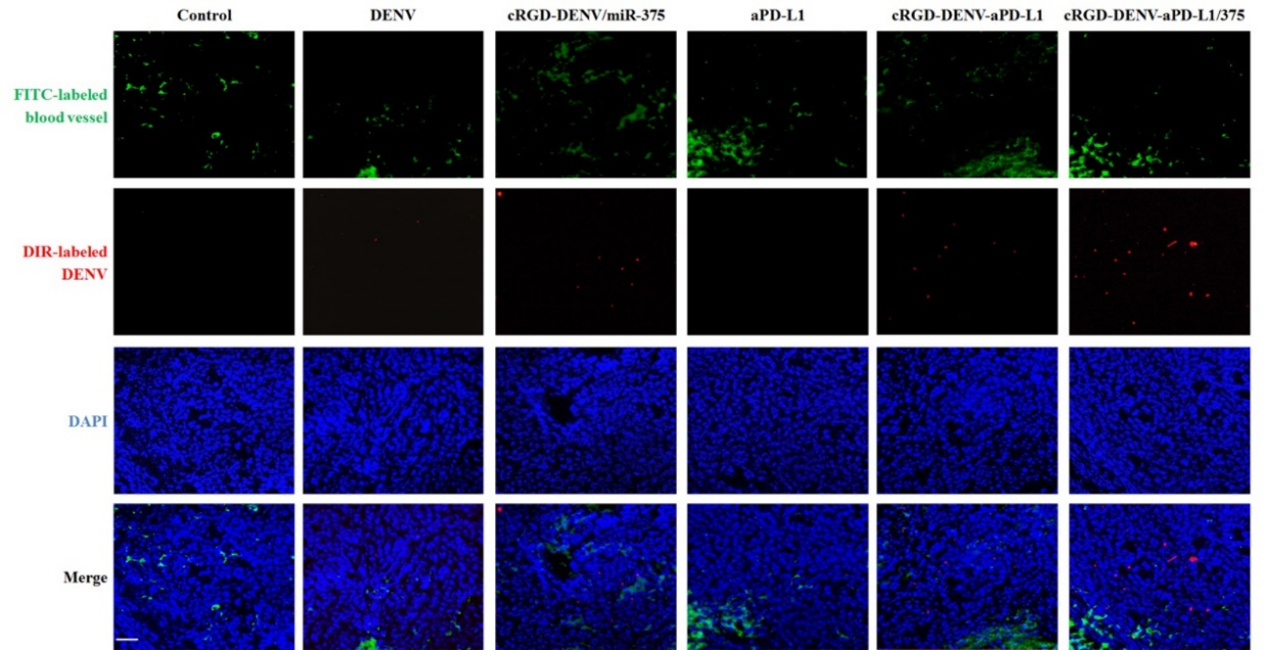


**Supplementary Fig.1** Tumor penetration analysis. BALB/c Nude mice bearing KYSE-150 tumors of ~200 mm^3^ were i.v. injected with saline, DENV, cRGD-DENV/miR-375, aPD-L1, cRGD-DENV-aPD-L1, cRGD-DENV-aPD-L1/miR-375. Ten minutes before sacrifice at 6 h postinjection of the conjugates, the mice were i.v. injected with FITC-labeled Lycopersicon esculentum lectin to stain the blood vessels. The tumors were excised and sectioned into 10 μm slices and observed by confocal microscopy. The DIR-labeled nanodrugs are shown in red, the blood vessels are shown in green, and the DAPI stained nucleus was shown in blue (Scale bars 50 μm).


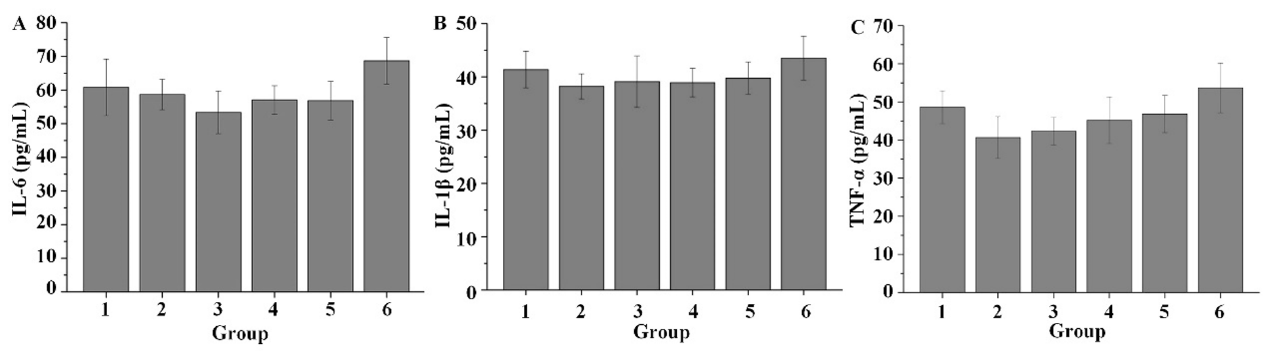


**Supplementary Fig.2** Serum levels of IL-6(A), IL-1β(B) and TNF-α(C).( Group1: Control, Group2: DENV, Group3: cRGD-DENV/miR-375, Group4: aPD-L1, Group5:cRGD-DENV-aPD-L1, Group6:cRGD-DENV-aPD-L1/miR-375).

**Supplementary Tab. 1** Gene specific primers sequences.

| Name of primer | Forward primer (5’ to 3’) | Reverse primer (5’ to 3’) |
| --- | --- | --- |
| RT-miR-375 | CTCAACTGGTGTCGTGGAGTCGGC | AATTCAGTTGAGGGTTTGTG |
| miR-375 | ACACTCCAGCTGGGGCGACGAGCCCCTCGCA | TGGTGTCGTGGAGTC |
| RNU6B | GCTTCGGCAGCACATATACTAAAAT | CGCTTCACGAATTTGCGTGTCAT |

**Supplementary Tab. 2** Serum levels of ALT, AST, CRE, BUN, WBC.

| Group Index | ALT(IU/L） | AST(IU/L) | CRE(μmol/L) | BUN(mmol/L) | WBC(10^9^/L) |
| --- | --- | --- | --- | --- | --- |
| Control | 53.02±1.49 | 79.88±5.76 | 14.88±1.23 | 2.96±1.65 | 6.54±4.94 |
| DENV | 53.72±4.78 | 82.68±0.94 | 14.97±2.45 | 2.09±0.29 | 6.21±0.52 |
| cRGD-DENV/miR-375 | 52.94±1.93 | 75.14±3.54 | 15.09±5.64 | 2.24±0.61 | 8.26±1.02 |
| aPD-L1 | 52.46±2.38 | 74.02±3.36 | 14.98±1.03 | 2.15±0.26 | 6.53±1.87 |
| cRGD-DENV-aPD-L1 | 55.21±4.99 | 73.04±1.93 | 17.90±4.08 | 2.36±0.54 | 5.93±2.01 |
| cRGD-DENV-aPD-L1/miR-375 | 52.54±1.22 | 73.65±0.52 | 16.67±2.05 | 3.16±0.62 | 6.33±2.72 |
